# A Recent Visit to Gheel

**Published:** 1862-01

**Authors:** 

**Affiliations:** One of the Commissioners in Lunacy for Scotland.


### A RECENT VISIT TO GHEEL.

By Dr. Coxe,

One of the Commissioners in Lunacy for Scotland.

We insert the following observations, written for and about to appear in the *Scotsman* newspaper, but which have been courteously forwarded to us, on account of their intrinsic merit, and as strongly illustrating and confirming some of the views given by Dr. Browne in his paper on "Cottage Asylums," recently published in this Journal. The problem of Gheel has not yet been worked out, but we have at last ascertained the state of the "seclusion and refractory wards;" which, in common with the whole tenor of the letter, appears to demonstrate the absence of a supreme medical authority interpenetrating and overruling every department, the absence of guarantees for the humane and *judicious* care of the insane, and some of the evils of the unrestrained liberty so much boasted of.

It is right to add that this letter is one of several valuable letters from the same pen, on Lunacy, and Asylum Colonies in France, and which have appeared in the course of the past vacation in the pages of the *Scotsman*. We trust that these letters will be collected together and published in a separate form.

"Gheel, September 27, 1861.

"The lunatic colony of Gheel has of late years attracted considerable attention, and not without reason, for, rightly studied, it is capable of affording a most important lesson in regard to the accommodation which should be provided for the insane. I say rightly studied, for the opinion which the visitor will form of Gheel will very much depend on the spirit in which the investigation is undertaken, and on the aspect in which the establishment is seen. There is little under the sun either all wholly good or all wholly bad, and, accordingly, any one who goes to Gheel determined, on the one hand, to see nothing wrong, or, on the other, to find nothing objectionable, cannot be regarded in the light of a trustworthy observer. First impressions may be true, but there is always a considerable risk of their being false; and a judicious inquirer will look at both sides of a question before venturing to express an opinion on its merits. An asylum superintendent would naturally object to a judgment being passed on his establishment simply from a visit to the refractory wards; and the director of Gheel would be equally entitled to call in question any opinion formed on a similar partial inspection of his domain.

"I do not know the precise extent of the commune of Gheel, but starting from the village as a centre a brisk walk of about an hour in any direction brings you to the confines of its territory. During the 'course,' to use a French expression, the land will be found to be of very variable quality. In some parts it is tolerably rich and productive, in others thin and poor. The best land occurs in the neighbourhood of the village and round the larger hamlets, and the poorest generally on the outskirts of the commune, but mostly in the district called Winkelomsheide, where it gradually passes into a miserable sandy heath, interspersed with pools of water, and presenting some scattered arable patches, the cultivation of which yields a precarious and scanty subsistence to an impoverished peasantry.

"Your readers are already acquainted with the general features of Gheel as a place for the care and treatment of the insane, but it may be useful briefly to recapitulate them. The distinguishing principle consists in distributing the patients, in numbers not exceeding four, in private dwellings, instead of gathering them together in special establishments, in groups occasionally comprising not less than fifteen hundred or two thousand persons. The commune of Gheel contains above 11,000 inabitants, and distributed among this population there were, on the 31st December, 1859, 800 lunatics. Of this number 676 were natives of Belgium, chiefly from the provinces of Antwerp, Brabant, and East Flanders; and 124 were foreigners, mostly Dutch. Of the same population 3312 were resident in the village, and 7894 in the landward part of the commune, some in scattered houses, but the greater part in hamlets, which occasionally contain several hundred inhabitants. The total number of houses was 1913; and the number in which patients were received 617, or about a third of the whole. Of these again 233, or rather more than a third, were in the village, and the remaining 384 in the hamlets and separate houses of the landward district. It will be seen, on comparing the number of patients with the number of houses receiving them, that as a general rule only one or two are placed in each house. Stated precisely, there is accommodation for one patient in 280 houses, for two in 297, for three in 32, and for four in 8. Of the occupants of these houses, 372 were peasants, 25 shopkeepers, 17 shoemakers, 16 carpenters, 8 bakers, 10 labourers, 32 tavern-keepers, 10 employés of the colony, 5 teachers, 10 'rentiers,' 8 lace-workers, 7 smiths, 5 barbers, and 9 clog-makers. Other patients, in numbers of one, two or three, are placed with masons, ropemakers, saddlers, tanners, dyers, &c. In fact, patients are received indiscriminately by all classes of the community. Of the total number, 515 were employed in one way or another; and 285, either from inability or caprice, did not engage in any kind of work. The chief sources of occupation were agricultural labour and household work. The former provided employment for 130 men and 36 women, and the latter for 58 men and 176 women. Of the remaining 37 men and 78 women, the males were chiefly occupied as tailors, shoemakers, carpenters, smiths, &c., and the females in sewing, knitting, and lace-making. A lunatic asylum in its ordinary signification is an extensive building in which the insane are collected, very frequently in large numbers.

Some have been sent for curative treatment, others from being dangerous, others from inability to extend to them proper care at home, and others again because they were found to be a nuisance or an inconvenience. An asylum, then, may be said to meet a public want, which presents itself in a variety of aspects, and under phases which modern habits of life are every day rendering more and more complicated. But is there no risk, it may be asked, of the welfare and happiness of the patient being sometimes sacrificed to the convenience or comfort of his family? or is it necessary or natural that the insane should, as a general rule, be all gathered together in large barrack-like buildings, and be subjected to the same wearisome routine and irksome discipline? No doubt the existing system has its advantages. It affords convenient means of withdrawing from the family circle those who are unfit for ordinary social life, and of placing them without much trouble or delay in circumstances in which their power to do harm will as much as possible be neutralized, and in which their eccentricities will not attract public attention. And there can be no doubt that the seclusion and isolation which asylums afford the means of enforcing, must in many cases greatly conduce to recovery, and therefore, both for the cure and for the care of the insane, they are calculated to confer inestimable advantages both on the patients and on society. But must asylum treatment, or rather what it often degenerates into—asylum confinement—be continued as long as the patient remains afflicted with insanity? What is expected from such prolonged seclusion? Is comfort increased, mortality lessened, or expenditure diminished? These are all vital questions in the disposal of the insane, and the experience derived from Gheel enables us in some degree to give them an answer. But Gheel, be it remembered, is not a model institution. Even its warmest advocates do not claim for it this position. But even with all its existing imperfections, it teaches us the important lesson that a large proportion of the insane can be properly cared for without falling back on the restraint and seclusion of an asylum. I am far from maintaining that all the patients who are sent to Gheel are proper subjects for such an establishment. Some are of dirty habits; others are dangerous, noisy, suicidal, or disposed to wander. Such patients are not only unsuitable inmates for private dwellings, but also improper subjects for an institution in which efficient restraint can be exercised only by mechanical contrivances. But the evils to which I here advert have long been recognised, and by no one are they more thoroughly admitted than by Dr. Bulckens, the present enlightened medical director of the colony. Many years ago, the necessity of providing an ordinary asylum in connexion with the colony of Gheel was urged upon the Belgian Government by the celebrated Esquirol. Representations to the same effect have been constantly made by the Belgian Lunacy Commissioners since their appointment; and at length, principally through the influence of the late Professor Guislain, of Ghent, the necessary funds were voted by the Chambers. The building is now ready for the reception of patients, and will be occupied early in spring. Unfortunately, however, it is on too small a scale to receive the whole of the patients who are not suitable for private care; and, indeed, its object is more that of an hospital for the treatment and observation of recent cases than a place of refuge for dirty and troublesome patients. The accommodation is calculated for fifty inmates only (twenty-five of each sex), of whom thirty-four will occupy associated dormitories, and sixteen be placed in single rooms. It is probable, however, that through the pressure for accommodation, the numbers in the associated dormitories will be considerably exceeded. The plan was furnished by Professor Guislain, whose views it embodies as previously carried into execution in the asylum of Ghent. Its characteristic features lie in the arrangement of the single rooms, and in the laying out of the airing courts. The single rooms are placed between two corridors, having the door opening into the one, and an unglazed window, provided with an ornamental iron frame, looking into the other. Facing this unglazed window, in the outer wall of the corridor is a glazed window, which overlooks the airing court. The advantages ascribed to this arrangement are the introduction of warmth through the unglazed window from the heated corridor, the free supply of light and fresh air, and the facility it affords of efficient surveillance. The chief peculiarity of the airing courts consists in small yards being attached to each of the 'strong' rooms, with the view of affording to its occupant the means of exercise without fear of being excited by other patients.

"The nature of the accommodation of the private dwellings varies in different parts of the commune according to the occupation and circumstances of the owners. In the village, the larger hamlets, and better class of farms, the houses are generally substantial erections of brick, with floors of deal or tiles; but in the remoter and poorer districts the walls are frequently of mud supported on wattle, and the floors of bare earth—damp, rough, and uneven, like those of Highland cottages. The comforts enjoyed by the patients vary in a like degree. In the landward parts of the commune the farm or croft contains on an average perhaps about ten acres. On inquiry, I have found its extent as great as thirty and as small as an acre and a half. The smaller possessions are chiefly where the land is poorest, and here also is generally found the greatest amount of poverty. The rent paid for about ten acres of fairish land, with a tolerable house, was stated to me to be 240*f*., or rather less than 10*l*.

"A Gheel crofter's house of the better sort is much larger than the dwelling of a small farmer in Scotland. The day-room or living-room, which is first entered, is generally of large size, with a huge projecting chimney, under which there is commonly sitting-room for several persons. Over the fire, which is always on the hearth, and of turf or peat, is usually suspended a large pot or cauldron, in which the food for the cattle is prepared. Plates and dishes of delf ware, with a goodly array of beer-stoups and glasses, are generally in sufficient abundance to fill a number of shelves fixed on the walls; and in almost every house there is an ancestral-looking eight-day clock. On one side of this room, and generally opposite the fireplace, is the byre, usually tenanted by from four to six cows; and on the other are the bed-rooms, and a sort of work-room containing baking-troughs, churns, &c. But bed-rooms occupied by patients or members of the family frequently occur in queer, out-of-the-way corners, occasionally reached by trap-stairs or ladders, resembling those attached to masons' scaffolds. The patients' bed-rooms are generally of small size, but they are all single. The furniture is scanty, but the bedding and general aspect of the rooms convey as favourable an impression as is derived from an inspection of those occupied by the family. On the whole, there is an appearance of rough comfort and plenty, and the looks of both the patients and their guardians give indication that the means of subsistence are adequate. Milk, butter, and cheese constitute the chief animal portion of their diet; and bread, potatoes, and vegetables of various kinds, its chief bulk. But in a number of houses I noticed bacon suspended from the roof, and other indications of fulness. The chief evil under which the Gheel houses suffer, especially those of the landward district, and more particularly of its poorer portions, appeared to me to consist in the insufficient means of warmth. In most of the houses there is a cooking-stove, which stands either in the living-room or in the adjoining work-room, and which diffuses a comfortable degree of heat; but the sleeping-rooms are frequently remote from its influence, and, being without fireplaces of their own, must in winter be extremely cold. Moreover, in the mud and wattle houses the walls are so thin, and frequently also in such a dilapidated condition, that the temperature there must fall extremely low. I am not surprised, therefore, to find that of the 257 deaths which took place in the four years 1856-59, 160 occurred in the six winter months, against 97 in those of the summer half-year.

"A visitor who should inspect merely the houses of the village, or of the larger hamlets, would be apt to form too high an opinion of the Gheel system of treatment; but, on the other hand, one who visited merely the remoter hamlets and the scattered cottages of the Winkelomsheide would infallibly fall into the opposite error. Herein, accordingly, may consist the explanation of the very different accounts which have been published of Gheel. But, in forming his opinion, the visitor must bear in mind that the patients are classified according to the very same principles which regulate classification in ordinary asylums. They are placed in groups or zones, which form the substitutes for asylum wards. Thus, in the village and larger hamlets are placed the quiet, better-behaved, and more industrious of the patients; while the more noisy, dirty, and least manageable are placed in the remoter hamlets, and in the separate houses of the Winkelomsheide. Several reasons combine to suggest this mode of classification. In the first place, in the village and the more populous landward districts, the public peace would be disturbed by noisy or troublesome patients, and decency outraged by the obscene; and, in the second place, the inhabitants of the village, and the more wealthy of the peasantry, are above the pecuniary necessity of receiving such patients. They accordingly refuse them as inmates, and indeed decline to retain any patients whose habits have become filthy; and thus, as a matter of necessity, all the worst patients, whether included in this category from being noisy, destructive, dangerous, filthy, or obscene, gravitate into the remotest districts and poorest houses. Such cases constitute the opprobrium of Gheel. But an erroneous idea would be formed of the system if it were imagined that the patients continued permanently in the houses in which they are first placed. On the contrary, their guardians are changed whenever this step seems advisable, either from the progress of the malady, from incompatibility of temper, or from any of the other numerous causes which, in the nature of the circumstances, must be constantly occurring. During the year 1859, accordingly, there were 132 changes of domicile.

"Of the 800 patients, 68 are subjected to some degree of mechanical restraint; but in 51 of these cases this is restricted to anklets connected by chains about a foot in length, which are worn to prevent escape. About fifteen patients wear leather girdles, to which their arms are attached by short chains in such a manner as to prevent the dangerous employment of their hands, without depriving them entirely of their use, and two require the strait-waistcoat. But the concentration of bad cases, consequent on the system of classification adopted, is apt to produce an unfavourable impression on the visitor, who, on finding in certain localities every third or fourth patient hobbled, or every sixth or eighth with his hands fastened in the manner described, might, on limited inquiry, be led to condemn the whole system; and I cannot deny that the condition of several of these patients was very miserable. Personal cleanliness, and cleanliness of clothing and bedding, are occasionally greatly neglected, but I am not prepared to say in a greater degree than is the case with the peasantry with whom they are placed, and who, from the causes to which I have adverted, are occasionally in a state of great poverty and misery. Indeed, it is their poverty alone that induces them to receive such inmates, and the pittance which is paid for their maintenance—from 6½d. to 7½d. a-day—must under such circumstances prove totally insufficient to afford the means of providing for their proper care. Every patient requiring mechanical restraint should be considered an improper case for Gheel. Repressive means of this kind are not only to be deprecated on their own account, but also for the tendency to believe in their necessity which they keep alive. But I am satisfied that much of the restraint now in use at Gheel might, with a little additional care and trouble, be dispensed with. The chains to the legs, and the girdles round the body are worn night and day; and the clothes of the patients are so fashioned as to permit of their undressing without the removal of the instruments of restraint. This permanent application of the means of coercion is quite uncalled for, and I firmly believe that a regulation calling for the removal of the chains and belts every night would soon lead to the discovery that in many cases they could be entirely dispensed with. Whenever it was found necessary to continue restraint, the patients should be removed from Gheel or placed in the asylum. Unfortunately, however, as I have already said, the new asylum is of much too limited extent to receive all the patients, who, from one cause or another, should not be left in private dwellings; and hence the evils to which I have been directing attention are not likely to be soon eradicated. At this season of the year (September) the condition of the patients is frequently aggravated by ague. In almost every house in the poorer districts which I entered, I found several of the inmates, both sane and insane, suffering from intermittent fever; and this, no doubt, often helped to give a darker hue to the picture.

"But, fortunately, there is a brighter side to turn to. Of the whole 800 patients, I should say that perhaps 700 are comfortably and happily placed; and we have herein evidence, which cannot be called in question, that a large proportion of the insane may very properly be accommodated in private dwellings, and be allowed to enjoy the pleasures derivable from ordinary social life.

"The patients at Gheel are not by any means all fatuous or imbecile. Of every 100 admitted, 13 are affected by melancholia, 42 with mania, 4 with monomania, 34 with dementia, and 7 with epilepsy. I state this fact as there seems to be a general impression that the cottage system is applicable only to the fatuous or demented. This, however, is very far from being the case, and at Gheel may be seen many patients affected with mania or melancholia who, I conceive, are there under more favourable circumstances than they would be in closed asylums: at the same time, I admit, I saw some cases, especially of patients suffering under the monomania of persecution, which I did not consider properly placed at Gheel. In a very large proportion of the admissions, the affection was already chronic and the prognosis unfavourable, only 17·6 per cent. being considered as affording much hope of recovery. Indeed, the greater portion of the patients have been brought from other asylums already in a state deemed incurable; the proportionally small number of curable cases being chiefly paupers of Brussels, which city sends, with few exceptions, the whole of its insane poor to Gheel. Accordingly, of the total number of patients, 216 were from this town. The position of Gheel as a place of curative treatment cannot therefore be inferred from the number of recoveries. Still it appears from the registers that 19 per cent. of the admissions during the four years 1856-59 were completely restored to sanity, and the advocates of the system argue that this proportion, considering the class of patients admitted, is extremely satisfactory. The deaths in 1858 amounted to 9 per cent. on the average numbers resident, and the average age at death during the four years just named appears to have been nearly fifty-three years. Of deaths from suicide there were only two in four years, and only one case of pregnancy. No death is recorded in this time from violence by a patient. To afford a standard of comparison, I may state that in the public asylums of Scotland, in the year 1860, the proportion of recoveries on the admissions was 37·7 per cent. for males, and 40·1 per cent. for females; the percentage of deaths on the average numbers resident, 10·2 for males, and 7·5 for females; and the average age at death, 44·5 years for males, and 9·42 years for females. The deaths from violence and suicide were more numerous than at Gheel, and immunity from pregnancy was certainly not less.

"In the medical administration of Gheel there is room for improvement. The duties of the director, M. Bulckens, are too multiform, and his time is too much occupied with correspondence and the keeping of registers to permit of sufficient personal inspection of the patients.

In stating this, I do not overlook the fact that each of the four sections into which the commune is divided has already its special medical inspector; but the proper working of the system must so much depend on the thorough acquaintance of its head with everything that is going on, that nothing should be allowed to interfere with frequent visitations by himself. Where the distances are so great, the assistance of a horse seems indispensable for the efficient discharge of this duty; and, unfortunately, the emoluments of M. Bulckens' office are too limited to afford him help of this kind. The talents and zeal of this gentleman are deserving of a much higher reward than is at present accorded them."

It may be interesting, and will carry up the literature of the subject to the present day, to append to the valuable observations of our countryman, Dr. Coxe, the impressions of Dr. Theobald Güntz, received during a visit of several days to Gheel in the year 1853, but which have just appeared in the last number of the *Allgemeine Zeitschrift für Psychiatrie und Psychish-gerichtliche Medicin.* Dr. Güntz's experience was obtained during the most palmy and unclouded days of our friend Dr. Parigot, whose enthusiasm was such as to carry away captive the inquirer, or, at all events, to reconcile him to some of the aspects of insane colonies. Dr. G. is first struck by the appearance of a man at an inn about a league from Gheel, decorated with feathers, cockade, ribbons, and paper orders, who was evidently insane. He was kept at a distance by the whip of the coachman, but soon became, by his loquacity and forwardness, the butt of the passengers. This painful impression was followed by another; for he encountered a patient whose hands were fastened by a chain to the wrist, and whose feet were secured by iron chains and fetters. "Had I not known where I was, I should have supposed myself in a convict colony." In visiting the cottages, Dr. G. was rejoiced to see the comfortable and happy appearance of many of the patients: on the other hand, however, he saw in many houses an iron ring fastened in the wall near the fireplace, here and there also a scourge, and even a chain and wristband hanging on the wall, the purpose of which might be guessed, but which was rendered clear by seeing an unfortunate creature confined by the hip, so that he could scarce move a step on either side. He likewise visited a dirty patient, and found him in a corner of the house, near two goats, lying upon straw, entirely naked, with no covering, bed, or bedding. Dr. G. on witnessing this scene openly and at once protested against such maltreatment. Dr. G. remarks that the physician's power is limited, that of the attendants great. The distribution of the patients is regulated by a committee; and as the whole village is divided into two parties, the liberal and the strictly catholic, the selection of the custodian does not depend so much upon their worthiness as upon their religious belief. A medical inspection takes place four times a year, and it depends upon the will of the attendant whether the physician is called in to see a critical case or not. It must be admitted that these guardians, by living constantly with the insane from their youth, have acquired a valuable knowledge and training: at all events, they confer great benefits upon their charges. After commenting upon the insufficient control, which was met by Dr. Parigot's observation that 10,000 sane superintended 1000 insane—upon the number of suicides and other accidents—upon the patients and their guardians speaking different languages,—Dr. Güntz left Gheel with the conviction that, while it possessed many advantages, it could not be regarded as a model. Dr. G. continues, that many improvements have been effected since his visit, but that these are due, not to internal developement, but to incentives from without. The percentage of cures has increased of late years—in fact, ever since the Gheel system, strictly so called, has been relaxed. While he admits that many small alleviations and recreations are more easily procurable in a family than in a large institution, he argues in favour of a judicious measure of restraint as a means of cure. He further objects to the crowding together of the patients in Gheel, and to the non-separation of the sexes; and then concludes that even now it is entirely unfitted to be a model for insane colonies, or as a substitute for institutions for the insane. He conceives that harmless incurables, and curables in a state of convalescence, are the classes of patients who might be placed in a colony, but that all others should be committed to an asylum; and approves of Dr. Roller's plan of a central asylum with radiating villages, to which patients should be removed according to their state of convalescence. He proposes that in these villages the sexes should be separated and the numbers limited.—*Ein Beitrag zur Frage über Irren Colonien, von Dr. Theobald Güntz*, p. 339.

"An attempt to reconcile the various accounts of this colony would be vain and profitless. They are not, in reality, conflicting: they are descriptions of the same institution at different periods; at different stages of development; by the inhabitants of different countries, accustomed to different modes of lodging and managing the insane poor. Insensibly, and with rigid adherence to truth, these narrations must be influenced by the education, the principles, the experience, even the feelings of their authors. It is quite obvious from all these sources, that a gradual approximation of opinions is going on; that the champions of sanguine and extreme and uncompromising views are disappearing from the field; that the inquirers bring away from Gheel convictions of the excellence and applicability of parts of the system pursued, and carry into the colony the knowledge of revolutions and improvements unheard of during its mediæval existence, and tried and sanctioned beyond its pale: and the expectation is suggested that in this its age of *renaissance*, there may grow up under our own observation, and partly through our own influence, that model so long craved as the successor to the ever-recurring plagiarism in stone and lime, the vast blockhouse; and which would unite in itself an asylum for chronic, an hospital for acute cases, and cottages for the tranquil and industrious."

